# Long-Term Efficacy of Carboxymethyl-Chitosan in Advanced Knee Osteoarthritis: A Twelve-Month Follow-Up Study on Non-Responders to Hyaluronic Acid

**DOI:** 10.3390/biomedicines13020270

**Published:** 2025-01-22

**Authors:** Nicola Manocchio, Carmelo Pirri, Concetta Ljoka, Andrea Sorbino, Nicolò Piacentini, Cristiano Monello, Giulia Vita, Calogero Foti

**Affiliations:** 1 Physical and Rehabilitation Medicine, Clinical Sciences and Translational Medicine Department, Tor Vergata University, 00133 Rome, Italy; nicola.manocchio@uniroma2.it (N.M.); cljoka@gmail.com (C.L.); a.sorbino@quaderni.biz (A.S.); npiacentini@yahoo.com (N.P.); cristiano.monello@gmail.com (C.M.); giuliavita55@gmail.com (G.V.); 2 Department of Neurosciences, Institute of Human Anatomy, University of Padua, 35121 Padua, Italy; carmelo.pirri@unipd.it

**Keywords:** knee osteoarthritis, intra-articular injections, carboxymethyl-chitosan, hyaluronic acid, quality of life, pain

## Abstract

**Background**: Knee osteoarthritis (OA) is a prevalent degenerative joint disease characterized by the degeneration of joint cartilage. Knee OA leads to pain, stiffness, swelling, and decreased mobility, significantly impacting the quality of life of affected people. Advanced-stage osteoarthritis often necessitates surgical intervention due to poor response to conventional treatments, such as intra-articular hyaluronic acid (HA). Carboxymethyl-chitosan (CM-C), an emerging therapeutic agent, has shown potential in reducing inflammation, improving lubrication, and enhancing joint function. This study aimed to evaluate the long-term efficacy of CM-C injections in patients with advanced knee osteoarthritis, non-responders to HA. **Methods:** This retrospective study included 16 patients (mean age: 79.56 years) with Kellgren–Lawrence grade 3–4 knee OA treated with a single intra-articular injection of CM-C. Pain and functional outcomes were assessed using the Visual Analogue Scale (VAS) and Knee Injury and Osteoarthritis Outcome Score (KOOS) at baseline (T0), one month (T1), three months (T2), six months (T3), and twelve months (T4). **Results:** Significant pain reduction was observed at early follow up, (VAS: T1 *p* = 0.0002, T2 *p* = 0.0265; KOOS Pain: T1 *p* = 0.0014). However, pain partially returned by T3 and T4. KOOS activities of daily living (*p* = 0.0005), QoL (*p* = 0.0396), and Sport and Free Time (*p* = 0.0367) subscales showed significant improvement at T1, though worsening trends were observed in subsequent follow up with raw values suggesting persistent benefits. Strong negative correlations were found between VAS and KOOS subscales at various follow ups. **Conclusions:** A single CM-C injection demonstrated early pain relief and functional improvement in advanced knee OA for non-responders to HA. However, the long-term effects may diminish over time, necessitating a careful consideration of re-treatment strategies or combined therapies.

## 1. Introduction

Osteoarthritis (OA) is a one of the most common chronic degenerative diseases affecting the musculoskeletal system, primarily characterized by joint pain and dysfunction. It significantly impacts patients’ functional status, leading to a progressive loss of autonomy in activities of daily living (ADL) and a deterioration in quality of life (QoL) [1].

In OA, affected joints undergo a series of degenerative changes, including the gradual breakdown of articular cartilage, thickening and hardening of the underlying bone, the formation of bone cysts and bone spurs (osteophytes), inflammation of the joint lining (synovium) or fluid sacs (bursa), thickening of the joint capsule, and potential damage to ligaments and menisci [2]. Articular cartilage is primarily composed of water and an extracellular matrix, with only a small percentage of cells called chondrocytes. These chondrocytes are responsible for producing essential components like proteoglycans and glycosaminoglycans. OA begins with disruptions to the normal repair and maintenance processes of articular cartilage. This leads to an imbalance, with increased production of some components and a rise in molecules that break down cartilage. Damage to the synovial lining often follows damage to the cartilage and bone. This synovial damage involves inflammation and thickening due to increased activity of synovial cells. This process results in changes to the synovial fluid, including increased production of a smaller type of hyaluronic acid [3]. OA commonly affects weight-bearing joints such as the knees and hips, leading to pain, stiffness, and reduced mobility. These symptoms contribute to difficulties in performing ADLs, and studies have shown that the intensification of OA manifestations correlates with a worse assessment of health and overall QoL, affecting physical, mental, and environmental domains [4]. Repetitive joint loading, common in certain occupations and high-impact sports, can increase the risk of OA. Prolonged exposure to mechanical stress accelerates cartilage wear and joint degeneration. Prior joint injuries, such as ACL tears or meniscal damage, significantly increase the risk of post-traumatic OA by disrupting joint biomechanics and initiating degenerative processes. Obesity is a major contributor to OA, increasing mechanical load on joints and promoting systemic inflammation. Women are more susceptible to OA than men, particularly after menopause, likely due to hormonal changes, anatomical differences, and biomechanical factors. Socioeconomic factors also play a role, as individuals with lower socioeconomic status often have limited access to healthcare and preventive measures, increasing their risk of developing and managing OA [5].

Knee OA is a particularly prevalent form of the disease, especially among older adults. It affects approximately 10% of individuals aged 60 years and older, with some studies indicating even higher prevalence rates depending on the population and geographic area studied [4,6]. A study conducted in Spain found that the prevalence of knee OA was 12.2% among individuals aged 60 to 90 years, higher in women (14.9%) compared to men (8.7%). Similarly, a study from China reported an overall prevalence of symptomatic knee OA at 14.6%, with higher rates observed in females (19.1%) compared to males (10.9%) [7,8]. The high prevalence of knee OA among the elderly population has significant implications for healthcare systems worldwide. It necessitates effective management strategies that include both pharmacological and non-pharmacological treatments to alleviate symptoms and improve patients’ QoL [9]. Several treatment options are available for people with mild-stage OA, including non-steroidal anti-inflammatory drugs (NSAIDs), corticosteroids, paracetamol, opioids, and viscosupplementation [i.e., intra-articular hyaluronic acid (HA) injection]. These treatments aim to alleviate pain and improve joint function and mobility [10,11,12,13]. However, in patients with advanced-stage OA [Kellgren–Lawrence (KL) grade 3–4] [14] these treatments may not be sufficient [15,16]. In such cases, surgical interventions like joint replacement are often considered to improve QoL. While effective, surgery carries inherent risks such as infection, high rates of hospital readmissions, and potential fractures around the prosthesis [17,18,19,20,21]. Given these limitations, identifying alternative conservative therapeutic strategies for patients who do not respond to conventional therapies seems crucial from a rehabilitation perspective.

Chitosan is a linear polymer that is both biocompatible and biodegradable, derived from the N-deacetylation of chitin. It possesses mucoadhesive, antioxidant, and antimicrobial properties, making it valuable in numerous medical applications [22]. The carboxymethylated form of the molecule is called carboxymethyl-chitosan (CM-C). CM-C is emerging as a promising therapeutic agent for the treatment of OA, offering several beneficial properties that address key aspects of the disease [23,24]: (i) CM-C has been shown to inhibit the breakdown of articular cartilage, which is a critical factor in the progression of OA. By preserving cartilage integrity, CM-C helps maintain joint function and delay disease progression. (ii) CM-C also promotes the differentiation of mesenchymal stem cells into chondrocytes, the cells responsible for producing cartilage. This property is particularly valuable for regenerating damaged cartilage and enhancing repair processes within the joint. (iii) CM-C seems to stimulate the production of type I and II collagen, supporting the structural integrity and resilience of cartilage and contributing to improved joint function. (iv) CM-C inhibits inflammatory cytokines and catabolic enzymes released by chondrocytes, reducing inflammation and tissue degradation within the joint. This anti-inflammatory action helps alleviate pain and slows down OA progression.

Additionally, CM-C demonstrates superior lubricating capacity compared to traditional cross-linked HA formulations, leading to significant improvements in joint mobility. The superior lubrication is attributed to its unique chemical structure. Unlike cross-linked HA, CM-C is a linear polymer that forms a highly viscous solution at physiological pH. This structure enhances its ability to coat and protect articular surfaces, reducing wear and tear during joint movement in both in vitro and ex vivo models; CM-chitosan reduced friction more effectively, which is crucial for restoring smooth joint articulation in OA-affected joints. The better physical lubrication and CM-C’s ability to maintain a healthier joint environment results in enhanced joint mobility [25,26].

In our previous pilot study, CM-C showed promising results in reducing pain and improving functional outcomes in patients with advanced OA. Specifically, pain reduction was observed one month post-injection, as measured by the visual analogue scale (VAS), along with improvements in functional outcomes assessed through the knee injury and osteoarthritis outcome score (KOOS). However, these benefits gradually diminished over time, with partial symptom recurrence at six months. Notably, raw scores for VAS and KOOS domains did not return to baseline levels even at later time points, indicating a sustained but reduced effect [27]. In this study, we aim to further explore the potential of CM-C as a promising treatment option for patients with knee OA non-responders to HA by expanding the number of patients and extending the follow-up period.

## 2. Materials and Methods

This study has a retrospective design. Data acquisition was carried out assessing clinical sheets of patients attending the Physical Medicine and Rehabilitation outpatient clinic at the Tor Vergata University Hospital, Rome, Italy.

The study examined data from patients who received intra-articular CM-C injections, using a formulation composed of 60 mg/3 mL of CM-C, which included 2% (*w*/*w*) CM-C in a phosphate buffer with an additional 3.5% sorbitol.

The patient data included in this study were from people who met the inclusion criteria during the period from September 2022 to November 2024. These criteria ensured that the study focused on a relevant and consistent patient population, particularly those who had not previously responded to HA. A larger sample size and longer follow up (12 months) were used to improve the previous analysis.

To gather data, a physiatrist with extensive experience in managing knee osteoarthritis and performing injection therapies administered various rating scales. These scales were used to evaluate the patients’ conditions at multiple intervals: initially at the time of injection (T0), followed by assessments one month later (T1), then at three months (T2), six months (T3), and finally twelve months (T4) post-treatment. This schedule of assessments was aligned with standard clinical practice to ensure comprehensive monitoring of patient outcomes over time.

The clinical protocol was conducted, recorded, and reported following the Good Clinical Practice guidelines and the Declaration of Helsinki [28] and approved by our institutional review board (Comitato Etico Territoriale “Lazio Area 2”). Before collecting data, an informed consent form was signed by all the participants [29].

### 2.1. Inclusion and Exclusion Criteria

To ensure consistency with our previous paper, we applied the same inclusion and exclusion criteria as follows.

Inclusion criteria:

- Male and female patients of all ages with advanced and symptomatic gonarthrosis (radiographic KL: grade ≥ 3);
- Patients previously unsuccessfully treated with intra-articular HA injections in the knee and subsequently treated at the same level with CM-C;
- Patients with a minimum 12-months follow-up who underwent scheduled clinical assessments at 1, 3, 6, and 12 months.

Exclusion criteria:

- Patients not treated with CM-C;
- Patients for whom rating scales were not completed.

### 2.2. Rating Scales

For the same reason stated above, we applied the same rating scales used previously.

The VAS [30] is a widely recognized tool for assessing pain levels. Developed by Scott and Huskisson, the VAS consists of a straight line, typically 100 mm in length, with the endpoints labeled “absence of pain” and “maximum pain”. Patients indicate their current pain intensity by marking a point on the line. The closer the mark is to either endpoint, the more it reflects the intensity of the pain experienced by the patient.

The KOOS [31] was used to evaluate functional outcomes. It is a self-administered questionnaire designed to assess symptoms reported in the knee joint. The KOOS consists of 42 items divided into five domains: symptoms, pain, activities of daily living, sports and free time, and quality of life. Each item is answered using a 5-point Likert scale, where 0 indicates no problems or difficulties, and 4 indicates severe problems or difficulties. Scores for each subscale are calculated separately using the formula: 100 − (score obtained × 100)/(maximum score). This results in a percentage score for each subscale, ranging from 0 (indicating severe disability) to 100 (indicating excellent condition).

### 2.3. Statistical Analysis

All data were initially entered into an Excel spreadsheet (Microsoft, Redmond, WA, USA) and statistical analysis was performed using GraphPad PRISM 8.4.2 (GraphPad Software Inc., San Diego, CA, USA). The resulting effect size was calculated by G Power 3.1 (Universität Düsseldorf: Psychologie) and interpreted according to Cohen’s kappa as small (d = 0.20), medium (d = 0.50), and large (d = 0.80). For VAS, the effect size was d = 1.675 in our previous pilot study [27], α error prob = 0.05, power: 1-β err prob = 0.95, and the total sample size was =6. Nevertheless, we could include a sample of 16 individuals in our group. The normality assessment was carried out using the Shapiro–Wilk test. Descriptive statistics were calculated, including measure of central tendency and their dispersion ranges using mean ± standard deviation (SD) to describe parametric data.

To compare changes over time in VAS and KOOS scores across different time points, repeated measures analysis was conducted by repeated measure ANOVA followed by Tukey’s multiple comparisons test to adjust for multiple testing. Differences between time points were considered statistically significant at a *p*-value of <0.05.

In addition, Pearson correlation coefficients were calculated to assess the relationship between VAS scores and KOOS subscales (pain, ADL, sport and free time, QOL). Correlation strength was interpreted based on the value of Pearson’s r, with significance set at *p* < 0.05. All tests were two-tailed.

## 3. Results

A total of 16 patients were enrolled according to the inclusion criteria (7 males, 44%; 9 females, 56%). The mean age was 79.56 ± 3.89 years (range: min. 73 years, max. 88 years).

**Figure 1:**
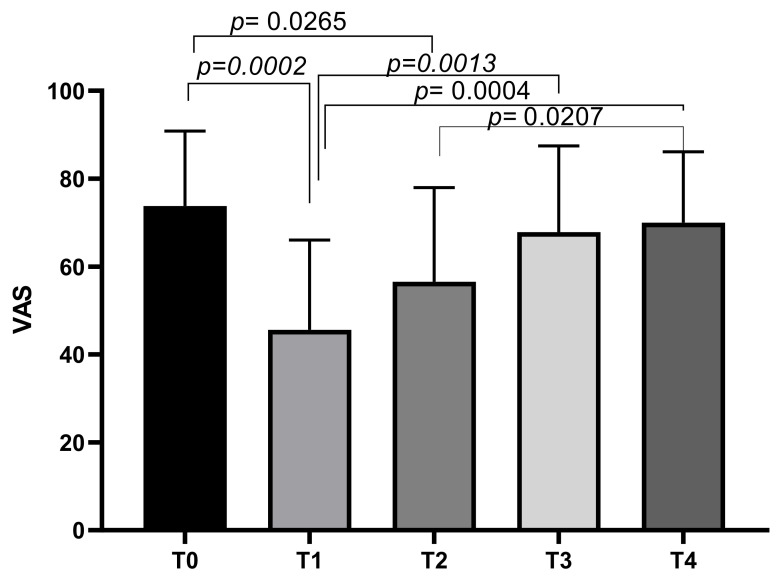
VAS score variations during study time points.

At T0, the mean VAS score was 73.75 ± 17.08, decreasing to a mean of 45.63 ± 20.4 at T1. The mean VAS score increased slightly at T2 (56.56 ± 21.43), T3 (67.81 ± 19.66), and T4 (70 ± 16.12). Tukey’s multiple comparisons test showed significant differences between VAS T0 and VAS T1 (*p* = 0.0002), and VAS T0 and VAS T2 (*p* = 0.0265). Other significant differences emerged between VAS T1 and VAS T3 (*p* = 0.0013), VAS T1 and VAS T4 (*p* = 0.0004), and VAS T2 and VAS T4 (*p* = 0.0207) (Figure 1).

**Figure 2:**
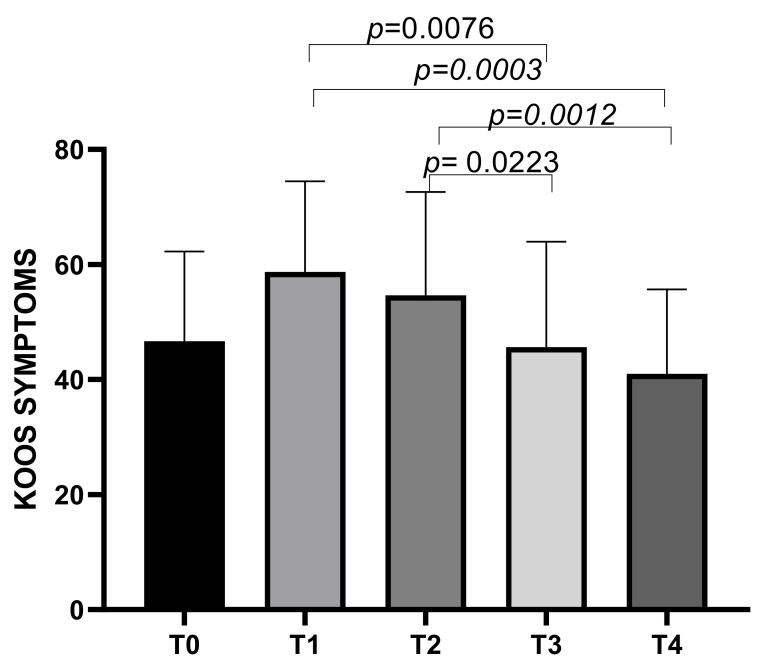
KOOS Symptoms score variations during study time points.

The KOOS symptoms subscale showed a mean score of 46.66 ± 15.61 at T0, increasing to a mean of 58.07 ± 15.74 at T1, then decreasing to means of 54.64 ± 17.96 at T2, 45.64 ± 18.31 at T3, and 40.99 ± 14.71 at T4. Statistically significant differences emerged between T1 and T3 (*p* = 0.0076), T1 and T4 (*p* = 0.0003), T2 and T3 (*p* = 0.0223), and T2 and T4 (*p* = 0.0012) (Figure 2).

**Figure 3:**
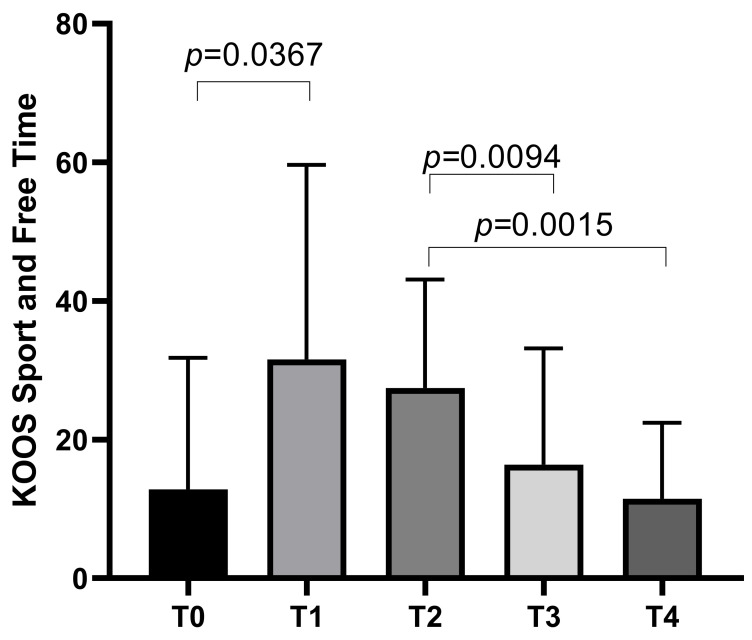
KOOS Sport and Free Time score variations during study time points.

The KOOS Sport and Free Time subscale started with a mean score of 12.81 ± 18.97 at T0, increasing to a mean of 31.56 ± 28.09 at T1 before decreasing to means of 27.44 ± 15.68 at T2, 16.38 ± 16.8 at T3, with a final mean of 11.44 ± 10.97 at T4. Statistically significant differences emerged between T0 and T1 (*p* = 0.0367), T2 and T3 (*p* = 0.0094), and T2 and T4 (*p* = 0.0015) (Figure 3).

**Figure 4:**
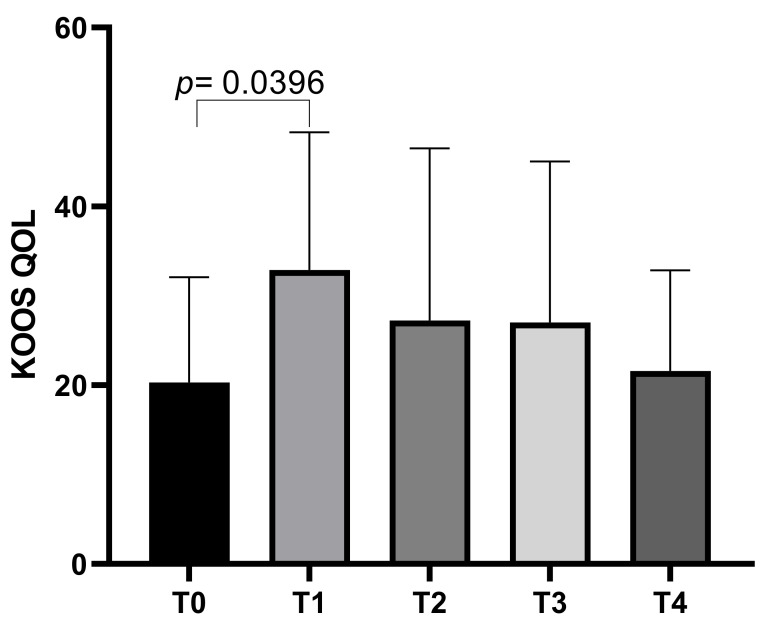
KOOS QoL score variations during study time points.

The KOOS QoL subscale showed an initial mean score of 20.31 ± 11.75 at T0, which increased to a mean of 32.88 ± 15.40 at T1 before decreasing slightly to means of 27.23 ± 19.26 at T2, 27 ± 18.01 at T3, and a final mean of 21.58 ± 11.25 at T4. Statistically significant differences emerged between T0 and T1 (*p* = 0.0396) (Figure 4).

**Figure 5:**
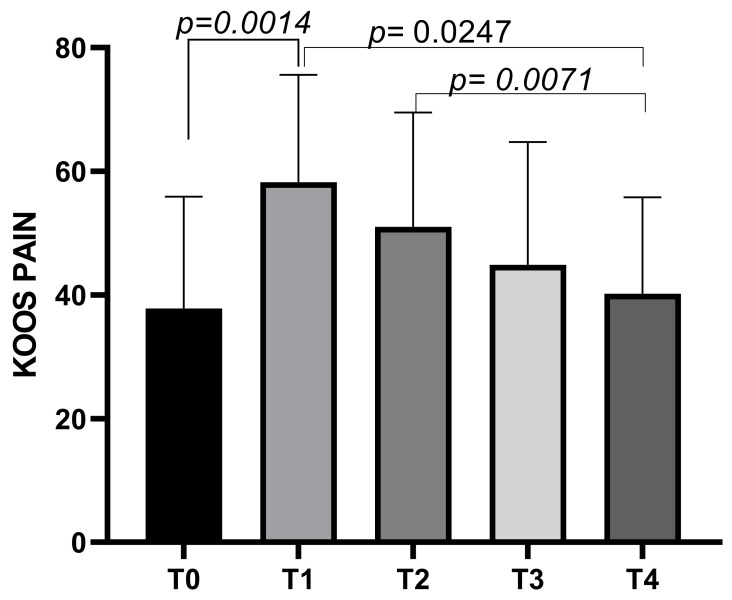
KOOS Pain score variations during study time points.

The KOOS Pain subscale had a mean score of 37.8 ± 18.08 at T0, increasing to a mean of 58.21 ± 17.42 at T1 before decreasing to means of 51.01 ± 18.5 at T2, and further decreasing to means of 44.86 ± 19.87 at T3 and finally to a mean score of 40.22 ± 15.59 at T4. Statistically significant differences emerged between T0 and T1 (*p* = 0.0014), T1 and T4 (*p* = 0.0247), and T2 and T4 (*p* = 0.0071) (Figure 5).

**Figure 6:**
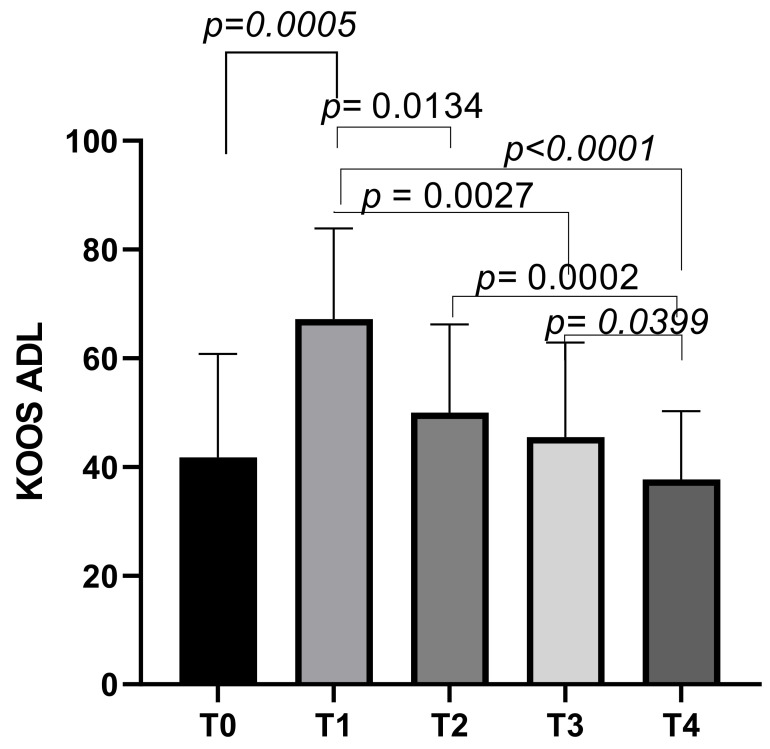
KOOS ADL score variations during study time points.

The KOOS ADL subscale had an initial mean score of 41.73 ± 19.04 at T0, which increased to a mean score of 67.21 ± 16.67 at T1 (*p* = 0.0030). The scores then decreased slightly to means of 49.99 ± 16.22 at T2, and further decreased to means of 45.52 ± 17.39 at T3. The final mean score was 37.68 ± 12.55 at T4. Statistically significant differences emerged between T0 and T1 (*p* = 0.0005), T1 and T2 (*p* = 0.0134), T1 and T3 (*p* = 0.0027), T1 and T4 (*p* < 0.0001), T2 and T4 (*p* = 0.0002), and T3 and T4 (*p* = 0.0399) (Figure 6).

### Correlation Analysis

Significant negative correlations were observed between VAS scores and specific KOOS subscales, indicating that reductions in pain were associated with improvements in functional outcomes (Table 1).

At baseline (T0), significant negative correlations were found between VAS and KOOS pain (r = −0.6165, *p* = 0.011) and VAS and KOOS Symptoms (r = −0.8159, *p* = 0.0001).

At the one-month follow-up (T1), negative correlations persisted between VAS and KOOS Pain (r = −0.6448, *p* = 0.007) and KOOS Symptoms (r = −0.6249, *p* = 0.0097).

By three months (T2), stronger negative correlations emerged across all domains, with significant relationships observed between VAS and KOOS pain (r = −0.6641, *p* = 0.005), symptoms (r = −0.6428, *p* = 0.0072), ADL (r = −0.5929, *p* = 0.0155), sport and free time (r = −0.6274, *p* = 0.0093), and QoL (r = −0.8129, *p* = 0.0001).

At six months (T3), negative correlations emerged again across all domains, with significant relationships observed between VAS and KOOS pain (r = −0.8034, *p* = 0.0002), symptoms (r = −0.8089, *p* = 0.0001), ADL (r = −0.6882, *p* = 0.0033), sport and free time (r = −0.8521, *p* < 0.0001), and QoL (r = −0.7383, *p* = 0.0011).

At the final follow up, twelve months (T4), negative correlations persisted between VAS and KOOS pain (r = −0.602, *p* = 0.0136), symptoms (r = −0.8143, *p* = 0.0001), ADL (r = −0.5703, *p* = 0.0211), sport and free time (r = −0.5765, *p* = 0.0194), and QoL (r = −0.5711, *p* = 0.0209).

## 4. Discussion

Following our pilot study [27] which showed promising results of CM-C in reducing pain and improving functional outcomes in patients with advanced OA, in this paper, we aimed to further investigate the effects of CM-C in a larger cohort of patients and considered a longer follow up period. Our updated findings demonstrate significant changes in pain and functional outcomes over time, as measured by the VAS and KOOS subscales. Notably, a significant reduction in VAS scores was observed between T0 and T1 and T0 and T2, indicating an early improvement in pain perception following the CM-C intra-articular injections. However, a subsequent gradual worsening was observed. While VAS scores improved significantly at T1 and T2, a significant decrease was observed comparing T3 and T4 to T1 and T4 to T2, suggesting a resumption of pain six months after treatment. On the other hand, it was interesting to find that at T4 (12 months) the VAS mean raw value was stable compared to T3, without statistically significant changes. The observed changes at the later time points (T3 to T4) indicate that while there is an initial reduction in pain, this relief may eventually plateau. This suggests that after a certain period, the rate of improvement in pain perception and functional outcomes slows down, stabilizing rather than continuing to improve significantly. Despite this plateau, the mean raw values at T4 were still slightly better compared to T0, underscoring the sustained benefits of CM-C over both short and long-term periods. This enduring improvement from baseline to 12 months post-treatment highlights CM-C’s potential as a long-lasting solution for pain management and functional enhancement. These findings are consistent with our previous report, which also demonstrated similar trends, and they have now been confirmed over a more extended period, reinforcing the reliability and effectiveness of CM-C as a therapeutic intervention [27].

In contrast to the significant changes observed in VAS scores, which primarily measure pain intensity, the KOOS subscales exhibited more variable results, reflecting the multifaceted nature of knee health. The KOOS is a validated self-administered questionnaire designed to evaluate various aspects of knee health, including pain, symptoms, ADLs, sport and recreation function, and knee-related QoL. It is widely regarded as a reliable and responsive tool for assessing changes in knee health across both short and long-term periods. The variability in KOOS subscale results may be attributed to the comprehensive nature of the questionnaire, which captures a broader range of outcomes beyond just pain relief. This makes it particularly suitable for tracking the effects of various treatments such as medication, surgery, or physical therapy. Moreover, it is effective in monitoring the progression of primary knee injuries or OA, providing a holistic view of a patient’s knee condition over time. The ability of KOOS to reflect subtle changes in different domains of knee health underscores its utility in both clinical and research settings for evaluating treatment efficacy and disease progression [32].

In our study population, the KOOS ADL subscale demonstrated a significant improvement between T0 and T1, indicating that patients experienced enhanced functional capacity for ADLs following treatment. This finding aligns with our previous report, reinforcing the notion that CM-C injections effectively aid in restoring basic functional abilities. However, a progressive worsening was observed at subsequent time points (T2 to T4), suggesting that while patients may quickly regain the ability to perform routine daily tasks, more complex activities might require a longer recovery period or additional rehabilitation efforts for significant improvement. This notion and the possible importance of rehabilitation in people with advanced OA is evident for sports-related functions and general perception of symptoms as well, as assessed by the KOOS sport and free time and the KOOS symptoms subscales. Notably, for all these subscales (ADL, symptoms, and sport and free time) mean raw values showed persistent stability till T4, highlighting the possible usefulness of retreatment at 6–12 months and association with other re-educational interventions after the first injection. This observation is consistent with other literature reports, which suggests that recovery of complex physical activities tends to take longer compared to basic functional abilities [33,34]. This could be due to the greater physical demands and coordination required for such activities, necessitating targeted rehabilitation to achieve full recovery. Interestingly, the KOOS QoL subscale showed statistically significant improvement at T1 compared to T0, with no significant worsening at later time points. Mean raw values were actually slightly better through T4 compared to T0. The first significant change and subsequent stability in our opinion suggests that while patients perceive initial benefits in QoL, measurable improvements may require more time or comprehensive interventions to be achieved.

A supporting finding in our study was the concordance between the VAS and the KOOS pain subscale at the one-month follow-up (T1). Both VAS scores and the KOOS pain subscale indicated a significant improvement in pain perception, despite the differences in how these tools measure pain. The VAS is a unidimensional measure focusing solely on pain intensity, providing a straightforward assessment of how much pain a patient feels. In contrast, the KOOS pain subscale encompasses a broader range of pain-related factors, including functional aspects of pain that may not change as rapidly or as noticeably in patients with advanced OA [35]. Parkes et al. have highlighted that one potential limitation of the KOOS pain subscale is its inclusivity of questions that might not be relevant to all patients, which can increase respondent burden and potentially reduce the sensitivity of the measure [36]. The positive outcome at T1 reinforces the notion of the usefulness of CM-C in producing positive effects in patients with severe OA non-responders to standard therapies. Moreover, the KOOS pain subscale showed improved raw scores through T4 compared to the time of injection, even in the presence of significant worsening comparing T4 to T1 and T2.

At T0, higher pain levels corresponded to worse functional outcomes as showed by the Pearson correlation. Notably, a strong negative correlation emerged between the VAS and various KOOS subscales at different follow-ups, suggesting a meaningful relationship between reduced pain intensity and improved functional outcomes. At earlier intervals, significant negative correlations between pain intensity (VAS) and functional outcomes suggest that effective pain relief is closely tied to improvements in daily activities and symptom management [37]. By T3 and T4, these correlations extend to broader domains such as sport and free time and QoL, indicating a sustained benefit of CM-C in enhancing overall knee function and patient well-being. These findings underscore the therapeutic potential of CM-C not only as a pain-relief agent but also as a means to improve functional capacity and QoL over time, particularly in patients who are unresponsive to conventional treatments like HA. The data further support the need for tailored re-treatment strategies or combination therapies to maintain these benefits in the long term.

On one hand, our findings suggest that carefully planning the timing of re-injection could enhance patients’ QoL; the observed partial return of symptoms at six and twelve months aligns with the natural progression of OA and the finite duration of intra-articular therapies. Periodic re-administration of CM-C could help maintain its anti-inflammatory, lubricating, and regenerative effects over time. Previous studies on other viscosupplementation agents, such as HA, have shown that repeated injections can prolong symptom relief and delay the need for surgical intervention [38,39]. Similarly, a tailored schedule for CM-C re-injections, potentially every six months, could optimize its benefits while minimizing disease progression. On the other hand, compared to our previous study, this is a stronger confirmation of the efficacy of CM-C in this peculiar population of patients that would otherwise require surgical intervention. Combining CM-C with physical therapy or other biomaterials like hyaluronic acid derivatives could provide synergistic benefits by addressing both mechanical and biochemical contributors to OA progression. Future research should prioritize optimizing treatment protocols through larger clinical trials while exploring potential synergies with regenerative therapies such as mesenchymal stem cells [40,41,42].

Moreover, CM-C demonstrates promising regenerative properties for OA. CM-C can modulate inflammation by inhibiting pro-inflammatory cytokines like TNF-α, IL-1β, IL-6, and IL-17, while stimulating the production of extracellular matrix components [43]. Preclinical studies have shown that chitosan-based hydrogels can effectively alleviate cartilage destruction and reduce pain compared to traditional HA [44]. Chitosan-based preparations have also shown to enhance bone deposition and to reduce osteoclast activity by modulating the OPG/RANKL signaling pathway. While this is primarily related to bone health, similar mechanisms could be leveraged for cartilage regeneration [45]. Furthermore, chitosan-based hydrogels exhibit sustained release properties, remaining in the joint environment for extended periods. This prolonged release can enhance therapeutic efficacy and reduce the need for frequent injections. Combining chitosan with other biomaterials, such as HA derivatives, can further optimize its therapeutic potential by synergistically improving lubrication and regenerative effects [44].

However, while preclinical studies are encouraging, rigorous clinical trials are necessary to validate the safety and regenerative efficacy of chitosan-based therapies in human patients with OA. Vibrotrography, a technique allowing assessment of the moving joint with a more accurate description, could be used for the analysis of improvement over time in future research [46,47].

To our knowledge, at the time of writing, three papers have been published regarding the role of CM-C in the treatment of patients with advanced OA non-responders to HA. The first two, by Emans et al. [48,49], focused on patients with knee OA KL grade 2–3 who were non-responders to simple oral analgesics (n = 95). This population differs significantly from our study, which included patients with more advanced (KL 3–4) disease. Despite these differences, both studies reported positive long-term effects of CM-C. Our findings further support the potential benefits of CM-C, particularly in patients with severe osteoarthritis who may not respond to conventional treatments.

The third study, by Lynen et al., similarly to Emans’ studies, involved 50 patients with knee OA KL grade 2–3. Additionally, Lynen et al. utilized the same outcome measures as our study, including the VAS and the KOOS [50]. Similar to our findings, Lynen et al. reported a rapid decrease in pain as assessed by VAS. Additionally, the authors observed improvements across all KOOS subscales in the majority of patients at the six-month follow-up.

In all three papers, as well as in ours, no serious adverse event was registered. Patients mainly reported cases of self-limiting arthralgia.

### Strengths and Limitations

Strengths and novelty of our study include the long-term follow-up (12 months) which provides valuable insights into the long-term effects of CM-C. Moreover, our study focused on patients with advanced-stage OA who were non-responsive to traditional HA treatments, highlighting the potential benefits of CM-C in a challenging patient population. These strengths contribute to the significance of our findings and provide valuable information for clinicians managing patients with advanced OA.

However, our study does not come without limitations. The first limitation is our relatively small sample size which may have reduced the statistical power of our analysis. It is challenging to recruit patients with advanced-stage OA who, given the limited availability of non-surgical options for severe disease, often lean towards surgical options without even considering alternatives. Moreover, VAS and KOOS may express subjective results that do not necessarily reflect objective changes in joint structure.

Increasing the sample size and transitioning to a prospective randomized controlled trial design could improve statistical power and minimize bias. Incorporating a control group treated with either placebo or alternative therapies could allow for direct comparisons and clearer attribution of outcomes to CM-C. Furthermore, extending follow-up periods could provide insights into long-term efficacy and safety. Investigating repeated injections at defined intervals may sustain therapeutic benefits over time, while combining CM-C with physical therapy or regenerative medicine approaches could yield synergistic effects. Finally, biomarker analysis and multi-center collaborations will help identify predictors of treatment response and enhance generalizability.

## 5. Conclusions

In conclusion, this study reinforces the potential of CM-C as an effective therapeutic option for managing advanced knee OA in patients unresponsive to HA. The findings demonstrate significant early improvements in pain relief and functional outcomes, particularly within the first month post-treatment. Strong negative correlations between pain intensity and functional measures further highlight the interconnected benefits of CM-C on both symptom alleviation and enhanced daily functionality. However, the gradual decline in efficacy observed at six and twelve months suggests that while CM-C provides sustained benefits compared to baseline, its therapeutic effects may diminish over time. This underscores the need for periodic re-administration or combination therapies to maintain long-term benefits.

The results also emphasize the importance of tailoring treatment strategies to address the multifaceted nature of knee OA. Future research should focus on optimizing re-treatment intervals, exploring synergistic effects with physical therapy or other regenerative approaches, and conducting larger-scale randomized controlled trials to validate these findings. Overall, CM-C presents a promising conservative treatment alternative for advanced OA patients, potentially delaying the need for surgical intervention and improving QoL.

## Figures and Tables

**Table 1:** Correlation results between VAS and KOOS subscales.

Time Point	VAS vs. KOOS Pain (r)	VAS vs. KOOS Symptoms (r)	VAS vs. KOOS ADL (r)	VAS vs. KOOS Sport and Free Time (r)	VAS vs. KOOS QOL (r)
**T0**	−0.6165 *	−0.8159 ***	−0.354	−0.2919	−0.4465
**T1**	−0.6448 **	−0.6249 **	−0.383	−0.47	−0.4057
**T2**	−0.6641 **	−0.6428 **	−0.5929 *	−0.6274 **	−0.8129 ***
**T3**	−0.8034 ***	−0.8089 ***	−0.6862 **	−0.8521 ***	−0.7383 **
**T4**	−0.602 *	−0.8143 ***	−0.5703 *	−0.5765 *	−0.5711 *

Significance Levels: * *p* < 0.05; ** *p* < 0.01; *** *p* < 0.001; more details in the main text. r: correlation coefficient; *p*: *p*-value.

## Data Availability

Data are available by reasonable request to the corresponding author.
